# Factors Influencing the Relationship Between Sugar Consumption and Depression Among Women Under Breast Cancer Treatment

**DOI:** 10.3390/bs15070940

**Published:** 2025-07-11

**Authors:** Yu-Chen Liu, Wen-Hung Kuo, Chiao Lo, Chiun-Sheng Huang, Meei-Shyuan Lee, Jen-Ho Chang, Chia-Chen Hsieh, Fei-Hsiu Hsiao

**Affiliations:** 1 School of Nursing, College of Medicine, National Taiwan University, Taipei 100233, Taiwan; 2 Department of Surgery, National Taiwan University Hospital, Taipei 110225, Taiwan; 3 Department of Surgery, College of Medicine, National Taiwan University, Taipei 100233, Taiwan; 4 School of Public Health, National Defense Medical Centre, Taipei 114201, Taiwan; 5 Department of Psychology, National Taiwan University, Taipei 106319, Taiwan; 6 Institute of Ethnology, Academia Sinica, Taipei 115201, Taiwan; 7 School of Nursing, College of Nursing, Taipei Medical University, Taipei 100233, Taiwan; 8 Department of Nursing, National Taiwan University Hospital, Taipei 110225, Taiwan

**Keywords:** depression, sugar consumption, mindfulness, quality of life, breast cancer

## Abstract

**Objective:** This study aimed to examine the mediating and moderating roles of quality of life, mindfulness, and savoring in the relationship between sugar intake and depression among women with breast cancer undergoing active treatment. **Methods:** This prospective observational study recruited seventy-eight women diagnosed with breast cancer within six months from a medical center in Taiwan. The participants completed patient-reported health surveys and dietary recalls. **Results:** Sugar consumption (*β* = 0.22, *p* < 0.01) and breast symptoms (*β* = 0.28, *p* < 0.01) were significantly associated with depressive symptoms. Simple mediation analysis indicated that quality of life fully mediated the relationship between sugar intake and depression (indirect effect = 0.15, 95% CI = 0.05 to 0.25), while breast symptoms played a partial mediating role (indirect effect = 0.10, 95% CI = 0.02 to 0.18). Moderated mediation analysis revealed that after-event savoring moderated the path between quality of life and depression (interaction effect = −0.04, 95% CI = −0.08 to −0.01). Furthermore, non-judgment mindfulness significantly attenuated the indirect effect of sugar consumption on depression via quality of life functions (moderated mediation index = −0.03, 95% CI = −0.06 to −0.00). **Conclusions:** Mindfulness and momentary savoring may act as protective factors, moderating the relationship between sugar consumption and depression. These findings support the proposal to integrate nutrition and mindfulness-based support into clinical care for women receiving active breast cancer treatment.

## 1. Background

Breast cancer is the most common cancer among women, with approximately 2.3 million patients diagnosed worldwide in 2022 (World Health Organization, 2024). While treatments significantly improve survival rates (Ricci-Cabello et al., 2020), depression remains a prevalent psychological burden during active treatment, affecting over 25% of women with early-stage (stage I–III) breast cancer. However, several studies have found that depression is a common kind of psychological distress observed in women with breast cancer undergoing active cancer treatment (Lan et al., 2022). Sugar consumption is used as a coping strategy to mitigate stress-induced depression. However, the overconsumption of sugar is linked to gut dysbiosis, oxidative stress, inflammation, insulin resistance, and other biological changes that increase depression risk (Reis et al., 2020). Yet, the role of sugar intake in depression during cancer treatment remains underexplored.

After diagnosis, many women with breast cancer adopt dietary changes to reduce recurrence risk (Mohd Zulkarnain & Vanoh, 2024; Shaharudin et al., 2013; Shin et al., 2024). Given the potential link between sugar intake and cancer progression (Birts et al., 2013; Epner et al., 2022), women with breast cancer may reduce sugar consumption accordingly (Depeint et al., 2018; Mohd Zulkarnain & Vanoh, 2024; Shaharudin et al., 2013; Tang et al., 2021). Stress activates the hypothalamic–pituitary–adrenal axis and increases cravings for sugary foods (Jacques et al., 2019). Accordingly, the stress of chemotherapy may increase craving for sugary foods from sweets, fruits, and natural fruit juices as a coping strategy. Some studies have found that patients reduce unhealthy added sugars but increase fruit consumption during chemotherapy (Pedersini et al., 2021), while others reported increased intake of both (Carlucci Palazzo et al., 2019). These findings suggest that during active treatment, breast cancer patients may increase sugar intake from both nutritious and non-nutritious sources.

Recent studies have examined the correlation between sugar consumption and quality of life among cancer patients during the post-treatment recovery phase. One study found that approximately 53% of women with early-stage breast or gynecological cancer reduced their sugar intake after diagnosis, and this reduction was associated with improved emotional function and less fatigue (Zainordin et al., 2020). Another study found that higher sugary food and drink consumption was linked to lower health-related quality of life (QoL) in all functional aspects and greater fatigue in colorectal cancer survivors up to 24 months post-treatment (Kenkhuis et al., 2023). These associations may be attributed to sugar’s pro-inflammatory effects, as chronic inflammation is a known contributor to depression, anxiety, and fatigue (Castro-Espin et al., 2023). However, there is limited research studying the relationship between sugar intake, depression, and QoL specifically during breast cancer treatment.

Dispositional mindfulness refers to an individual’s concentration on the awareness of their present moment experiences without reactivity or judgment (Kabat-Zinn, 2019; Meng et al., 2020). The Mindfulness-to-Meaning Theory proposes that mindfulness can enhance cancer patients’ QoL and reduce emotional distress through a series of mechanisms, including attentional control, positive reappraisals, savoring, and ultimately achieving better well-being (Garland et al., 2017). Mindfulness-based interventions have been shown to improve QoL and reduce depression among Chinese women with breast cancer undergoing chemotherapy (Wang et al., 2023; Zhu et al., 2023). Similarly, a recent scoping review found that mindfulness and yoga interventions improved both psychological well-being and metabolic outcomes in individuals with diabetes, evidencing the protective role of mindfulness practice (Cangelosi et al., 2024). Momentary savoring is defined as the capacity to attend to and appreciate positive experiences in the present moment, thereby enhancing emotional well-being (Bryant & Veroff, 2007). According to the broaden-and-build theory (Fredrickson, 2004), positive emotions elicited through savoring can broaden an individual’s thought–action repertoire and build personal resources, which in turn support coping with cancer-related symptoms. In Chinese cancer patients, momentary savoring was found to moderate the relationship between physical symptoms (e.g., fatigue, nausea) and depression (Hou et al., 2017).

In summary, stress-induced sugar consumption may increase depression risk, and its impact on QoL could mediate this relationship. However, evidence specific to women undergoing active breast cancer treatment remains limited. Mindfulness and savoring may buffer these effects. Therefore, the aims of this study are (1) to examine the mediating effect of QoL on the relationship between sugar intake and depression, and (2) to assess whether mindfulness and savoring moderate this pathway.

## 2. Methods

### 2.1. Study Design and Setting

This prospective observational study was approved by the hospital’s institutional review board (202201021RIND) and was reported in accordance with the STROBE (Strengthening the Reporting of Observational Studies in Epidemiology) guidelines (von Elm et al., 2007). Women with breast cancer were recruited from the outpatient breast center of a medical center in Taiwan. During routine visits, research staff approached eligible patients, who provided written informed consent. They completed self-administered questionnaires either at the clinic or at home, returning them by mail within a week. Research staff then conducted three non-consecutive 24 h dietary recall interviews via telephone over the following month. All data were collected within six months post-diagnosis while patients were actively receiving treatment.

### 2.2. Participants

The inclusion criteria were women aged 20 and older, within six months after being diagnosed with breast cancer, with no breast cancer recurrence, without other cancer, and under cancer treatment (mastectomy, chemotherapy, or radiotherapy). Patients diagnosed with stage IV cancer or who were unable to read questionnaires were excluded from this study. Of the 311 invited patients, 78 completed the study, 74 were ineligible, 127 declined, 28 lost contact, and 3 refused dietary recalls.

### 2.3. Measures

#### 2.3.1. Depressive Symptoms

We measured depressive symptoms using the Patient Health Questionnaire-9 (PHQ-9), which is a validated self-administered tool designed to assess the severity of depression by evaluating nine DSM-IV criteria over the past two weeks (Spitzer et al., 1999; Yeung et al., 2008). Each item is rated on a 4-point Likert scale, resulting in a total score ranging from 0 to 27, where higher scores indicate greater depression severity.

#### 2.3.2. Quality of Life

QoL was assessed using the European Organization for Research and Treatment of Cancer Quality of Life Questionnaire Core 30 (QLQ-C30) and the breast cancer-specific QLQ-BR45. The QLQ-30 measures global health status, evaluating functional scales (physical, role, emotional, cognitive, and social functioning), and various symptoms (fatigue, pain, nausea, vomiting, dyspnea, insomnia, appetite loss, constipation, and diarrhea), as well as financial status (Chie et al., 2003). The QLQ-BR45 consists of 45 items assessing breast cancer-related functions (body image, future perspective, sexual functioning, breast satisfaction) and symptoms (therapy side effects, hair loss distress, arm and breast symptoms, endocrine-related symptoms) (Bjelic-Radisic et al., 2020). Higher function scores indicate better functioning, while higher symptom scores reflect greater symptom severity.

#### 2.3.3. Five Facets of Mindfulness

The 15-item Five Facets of Mindfulness Questionnaire (FFMQ-15) was used to measure mindfulness skills across five facets: describing, acting with awareness, non-judgment of inner experience, and non-reactivity to inner experience in daily life (Baer et al., 2008). The participants responded to each item on a 5-point Likert scale, with each facet yielding a subtotal score ranging from 5 to 15. Higher scores indicate a greater tendency to engage in mindfulness practices in daily life.

#### 2.3.4. Momentary Savoring

Momentary savoring was measured by a 4-item Momentary Savoring Scale (MSS) based on a 5-point Likert scale (Jose et al., 2012; Sato et al., 2018). MSS measures counting blessings, sensory–perceptual sharpening, strategies for sharing with others, and experiential adsorption. The sum of the first two items measures during-event savoring, and the sum of the last two items assesses after-event savoring. Each subscale yields a score ranging from 2 to 10, with higher scores indicating greater momentary savoring.

#### 2.3.5. Energy Intake and Sugar Consumption

The Automated Self-Administered 24-Hour Dietary Assessment Tool (ASA-24), a validated web-based platform, was used to assess participants’ dietary intake (National Cancer Institute, 2024). Due to the lack of a Chinese language interface and limited culturally appropriate food items (Beasley et al., 2022), diet recalls were conducted through telephone interviews by a trained interviewer. The tool provided data on total energy intake and macronutrient composition (carbohydrates, proteins, and fats). Macronutrient percentages were calculated using standard energy values (4 Kcal/g for proteins and carbohydrates, 9 Kcal/g for fats). Minor discrepancies in ASA-24 calculations may slightly exceed 100%. Sugar consumption was analyzed, and its percentage of total energy intake was calculated to illustrate its energy contribution. To account for potential confounding by total energy intake, we applied the residual method (Willett, 2012) to estimate energy-adjusted sugar intake.

#### 2.3.6. Demographic and Clinical Data

Demographic data, including age, education, employment, and income, were collected via a structured survey. Physical status (height, weight, BMI, comorbidities) and cancer-related data (stage, treatments: mastectomy, chemotherapy, radiotherapy) were also recorded.

### 2.4. Data Analysis

Descriptive statistics represented by means, standard deviations, and frequency (%) served to describe the demographic of the sample population and the study variables. Zero-order correlations were assessed by using Pearson’s correlation analysis. Multiple linear regression analysis was conducted to examine the relationships between the potential factors and depression, examining the impact of multiple variables simultaneously. For mediation analysis, we applied Model 4 of Hayes’s PROCESS macro (version 4.2) for SAS 9.4 (Hayes, 2022). A significance level of *p* < 0.05 was adopted, and 95% confidence intervals (CIs) were reported for the indirect effects to provide a robust understanding of the mediation pathways. To minimize reporting bias, validated instruments were used, and all dietary recalls were conducted by trained interviewers. Complete-case analysis was applied, and no data imputation or sensitivity analyses were performed.

## 3. Results

### 3.1. Characteristics of Participants

In Table 1, it can be seen that 78 women with breast cancer participated this study with a mean age of 49.95 years (SD = 9.80, range 32−78) and with a mean BMI of 22.70 (SD = 3.24, range 18.13−35.34); most education levels were undergraduate and above (84.62%), and 55 women were employed (70.51%). For clinical characteristics, more than half (51.28%) were diagnosed with stage II. Of the 57.69% of participants who received surgery, 46.67% received breast-conserving surgery, and 53.33% received modified radical mastectomy. Most participants underwent chemotherapy (79.49%), and 35.90% had comorbidities.

### 3.2. Characteristics of Outcomes

The mean depression score was 6.45 (SD = 4.83), and 17.95% (n = 14) reached the clinical depression level (scores of 10 or greater). The mean score of the QLQ-C30 functions was 75.52 (SD = 14.26), and its’ highest sub-function domain was physical function (mean = 84.95, SD = 13.04), followed by role function (mean = 77.56, SD = 23.08) and cognitive function (mean = 76.92, SD = 21.19), with the lowest in social function (mean = 63.68, SD = 24.58). The mean score of QLQ-C30 symptoms was 25.16 (SD = 14.39), and the three most common symptoms were fatigue (mean = 35.61, SD = 18.81), insomnia (mean = 34.19, SD = 29.41), and financial difficulties (mean = 32.91, SD = 32.89).

The mean score of QLQ-BR45 functions was 29.39 (SD = 14.49), and the highest function was body image (mean = 62.71, SD = 27.71), followed by future perspective (mean = 39.32, SD = 29.79) and sexual enjoyment (mean = 22.22, SD = 16.27). The mean score of QLQ-BR45 symptoms was 26.85 (SD = 14.20). The most common breast cancer symptoms were upset caused by hair loss (mean = 57.07, SD = 36.41), systematic therapy side effects (mean = 37.79, SD = 18.71), and arm symptoms (mean = 22.65, SD = 24.12). The mean total score for mindfulness was 54.08 (SD = 7.02), with subscale scores detailed in Table 2. The mean scores of momentary savoring during and after events were 7.17 (SD = 1.26) and 8.36 (SD = 1.24), respectively.

For the nutrients of intake during active cancer treatments, the total energy was 1649.01 kcal (SD = 359.54), and the mean of percentages of energy of protein, total fat, carbohydrate, and total sugar were 25.62%, 36.28%, 44.73%, and 14.61%, respectively. Based on the recommended sugar intake for cancer patients, around 17% of the participants had sugar intakes of less than 10% energy, and 38% had less than 50 g sugar intake per day (Table 2).

### 3.3. Bivariate Correlation and Multivariate Regression Analysis

The correlations between the study variables are provided in Table S1. QLQ-C30 and QLQ-BR45 functions and symptoms, total mindfulness score, and savoring after event were significantly correlated to depression. Sugar intake was significantly correlated to depression, QLQ-C30 functions and symptoms, and QLQ-BR45 symptoms as well.

In Table 3, the univariate model shows that both grams of sugar intake and % of energy from sugar intake were significantly associated with depression. When controlled for stage, QLQ-C30 and QLQ-BR45 functions and symptoms, mindfulness, and savoring after events, only QLQ-C30 functions, QLQ-BR45 functions, and savoring after events significantly were associated with depression. Both grams of sugar intake and % of energy from sugar intake were significantly associated with depression in the multivariate linear regression models.

### 3.4. Simple Mediation Analysis

The findings of a simple mediation analysis revealed that QLQ-C30 functions, QLQ-C30 symptoms, and QLQ-BR45 symptoms all serve as mediators in the relationship between sugar intake and depression among women with breast cancer. Specifically, QLQ-C30 functions and symptoms play a significant mediating role in the association between sugar intake and depression, while QLQ-BR45 symptoms play a partial mediating role (Table S2).

### 3.5. Moderated Mediation Analysis

#### 3.5.1. The Moderating Effect of After-Event Savoring

Significant direct effects were observed between the QLQ-C30 functions and depression, as well as after-event savoring and depression. The interactions between the QLQ-C30 functions and after-event savoring were found to be significant (effect = 0.06, 95% CI = 0.03, 0.10). Additionally, the index of moderated mediation was significant for after-event savoring (indirect effect = −0.07; 95% CI = −0.12, −0.03). The conditional indirect effect for women with breast cancer with lower after-event savoring (indirect effect = 0.32; 95% CI = 0.16, 0.48) was found to be stronger than that for women with after-event savoring (indirect effect = 0.14; 95% CI = 0.05, 0.25).

In terms of the role of after-event savoring in the relationship of QLQ-C30 symptoms as a mediator between sugar intake and depression, we found that after-event savoring plays a moderating role between QLQ-C30 symptoms and depression. The interactions between the QLQ-C30 symptoms and after-event savoring were significant (*β* = −0.04, 95% CI = −0.08, −0.01). The index of moderated mediation was also significant for after-event savoring (indirect effect = −0.05; 95% CI = −0.09, −0.01). The conditional indirect effect for women with breast cancer with lower after-event savoring (indirect effect = 0.27; 95% CI = 0.11, 0.43) was found to be stronger than that for women with higher after-event savoring (indirect effect = 0.15; 95% CI = 0.06, 0.27).

**Figure 1:**
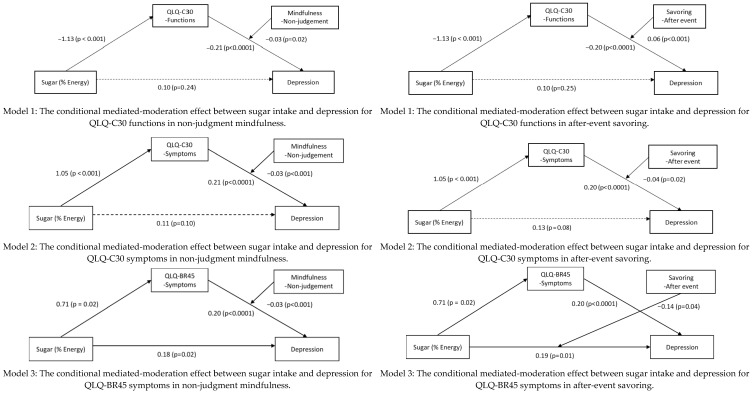
Mediated-moderation models (note: QLQ-C30, Quality of Life Questionnaire for Cancer; QLQ-BR45, Quality of Life Questionnaire for Breast Cancer). Solid arrows indicate statistically significant paths (*p* < 0.05); dashed arrows indicate non-significant paths (*p* ≥ 0.05).

**Figure 2:**
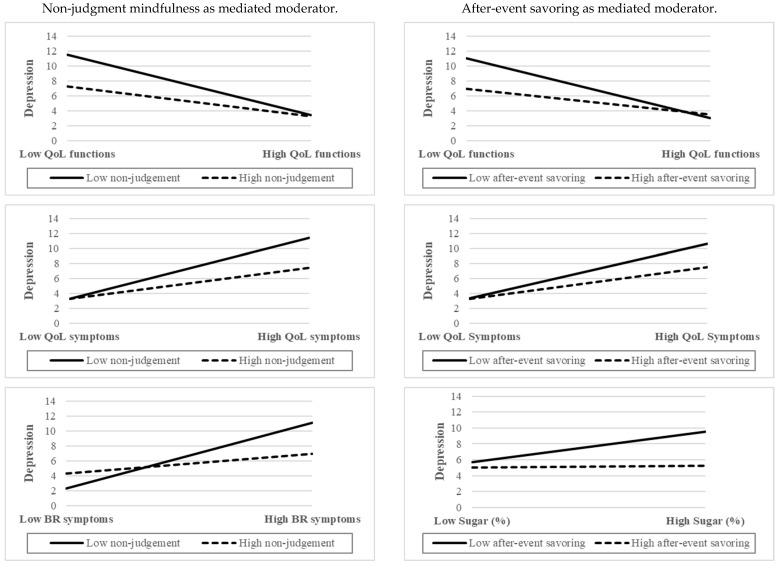
Interaction plots of mediated moderators with depression measured by PHQ-9 (note: QoL, quality of life; BR, breast).

In examining the role of after-event savoring in the relationship between QLQ-BR45 symptoms, sugar intake, and depression, our study revealed that after-event savoring acts as a moderator between sugar intake and depression. The interactions between sugar intake and after-event savoring were significant (*β* = −0.14, 95% CI = −0.27, −0.01). The index of moderated mediation was significant for after-event savoring (indirect effect = 0.14; 95% CI = 0.03, 0.26). The conditional indirect effect for women with breast cancer with lower after-event savoring (indirect effect = 0.36; 95% CI = 0.16, 0.57) was found to be stronger than that for women with higher after-event savoring (indirect effect = 0.02; 95% CI = −0.22, 0.26). The conditional indirect effect for women with breast cancer with lower after-event savoring was stronger than that for women with higher after-event savoring among above three moderated mediation models (Figure 1 and Figure 2).

#### 3.5.2. The Moderating Effect of Mindfulness

Significant direct effects were found between the QLQ-C30 functions and depression, as well as between non-judgment mindfulness and depression. The interactions between the QLQ-C30 functions and non-judgment mindfulness were significant (effect = 0.03, 95% CI = 0.00, 0.05). The index of moderated mediation was significant for non-judgment mindfulness (indirect effect = −0.03; 95% CI = −0.06, −0.00). The conditional indirect effect for women with breast cancer with lower non-judgment mindfulness (indirect effect = 0.32; 95% CI = 0.15, 0.51) was found to be stronger than that for women with higher non-judgment mindfulness (indirect effect = 0.16; 95% CI = 0.06, 0.27).

In terms of the role of non-judgment mindfulness in the relationship of QLQ-C30 symptoms as a mediator between sugar intake and depression, we found that non-judgment mindfulness has a moderating effect between QLQ-C30 and depression. The interactions between the QLQ-C30 symptoms and non-judgment mindfulness were significant (effect = −0.03, 95% CI = −0.04, −0.01), and the index of moderated mediation was also significant for non-judgment mindfulness (indirect effect = −0.03; 95% CI = −0.05, −0.01). The conditional indirect effect for women with breast cancer with lower non-judgment mindfulness (indirect effect = 0.30; 95% CI = 0.14, 0.47) was stronger than that for women with higher non-judgment mindfulness (indirect effect = 0.15; 95% CI = 0.06, 0.25).

Similarly, in the context of QLQ-BR45 symptoms, non-judgment mindfulness was also found to have a moderating effect between QLQ-BR45 symptoms and depression. The interactions between the QLQ-BR45 symptoms and non-judgment mindfulness were significant (effect = −0.04, 95% CI = −0.06, −0.02). The index of moderated mediation was also significant for non-judgment mindfulness (indirect effect = −0.03; 95% CI = −0.05, −0.01). The conditional indirect effect for women with breast cancer with lower non-judgment mindfulness (indirect effect = 0.22; 95% CI = 0.04, 0.39) was found to be stronger than that for women with higher non-judgment mindfulness (indirect effect = 0.07; 95% CI = 0.01, 0.15). The conditional indirect effect for women with breast cancer with lower non-judgment mindfulness was stronger than that for women with higher non-judgment mindfulness in more than three moderated-mediation models (Figure 1 and Figure 2).

## 4. Discussion

### 4.1. Key Findings and Interpretation

This study examined how sugar consumption, QoL, mindfulness, and savoring interact to influence depression in breast cancer patients. Our findings on the association of high sugar intake with increased depression risk were consistent with a previous study (Reis et al., 2020). Kenkhuis et al. (2023) also found that a higher intake of sugar-sweetened drinks was linked to lower QoL functions and greater fatigue in colorectal cancer survivors. Our study found that both the general QOL fully mediated the relationship between sugar intake and depression, while breast cancer-specific symptoms played a partial mediating role. Our study findings demonstrated that a higher sugar intake could affect QOL, which subsequently results in depression. An adequate caloric intake is recommended during active cancer treatment (American Cancer Society, 2024). Our findings suggest the that high sugar consumption may adversely affect QoL and further increase the risk of depression in women with breast cancer.

This study found that the non-judgment aspect of mindfulness played a mediated moderating role in the connection between sugar intake and depression, while after-event savoring acted as a moderator only when breast cancer symptoms mediated the relationship. Consistent with our findings, Jovaišaitė (2024) found that non-judgmental mindfulness strongly reduces psychological distress by decreasing self-critical thinking, interrupting negative thought cycles, and fostering emotional resilience. Medvedev et al. (2021) identified non-judgment as the core mindfulness facet associated with reduced distress and improved emotion regulation. Barcaccia et al. (2019) further found that non-judgmental mindfulness is the strongest predictor of both depression and anxiety. Individuals who judge their inner experiences tend to experience greater emotional distress, whereas adopting a normalizing, non-critical perspective is central to the psychological benefits of mindfulness. This mindset has also been shown to partially mediate the relationship between perceived maternal invalidation and expressive suppression.

Notably, only low levels of after-event savoring significantly amplified the indirect effect of sugar intake on depression, suggesting a moderated mediation pattern. Similarly, Chen et al. (2024) found that savoring beliefs enhance psychological resilience and reduce anxiety and depression. Although savoring involves both during- and after-event components, our findings suggest that only the latter is closely associated with depression. This supports Bryant and Veroff’s (2007) concept of “rosy expectation” and “rosy retrospection,” where individuals derive greater enjoyment from anticipating or recalling positive events than from experiencing them in the moment.

Furthermore, after-event savoring involves sharing experiences with others and engaging in reflection, which enhances appreciation for past events (Bryant & Veroff, 2007). Through social sharing, individuals relive positive moments from the perspective of loved ones, amplifying positive emotions. Complementing the social sharing, savoring denotes a state of deep immersion and engagement with the positive experience. This intrapersonal dimension of savoring facilitates a subjective slowing of time and prolonged positive affect. Research has shown that the inability to savor positive experiences is associated with increased risk of depression (Bryant & Veroff, 2007). Individuals who struggle to share their positive experiences with others or to fully immerse themselves in the memory of these events may be more susceptible to depressive symptoms. Conversely, the ability to effectively savor positive events can serve as a protective factor against depression by enhancing positive affect, fostering social connections, and promoting a sense of meaning and well-being.

### 4.2. Perspective for Clinical Practice

Our findings offer meaningful implications for clinical care and future research on the mental health of women with breast cancer. Healthcare providers could incorporate nutritional counseling into oncology care, with attention to reducing sugar intake to improve QoL and alleviate depressive symptoms (Kenkhuis et al., 2023; Zainordin et al., 2020). Integrating mindfulness-based interventions and savoring techniques may enhance emotional regulation and foster resilience during treatment (Chen et al., 2024; Wang et al., 2023).

### 4.3. Limitations

The limitations of this study should be acknowledged to contextualize the findings appropriately. First, the analysis of breast cancer-related functional QoL was hindered by missing data, which may have affected the robustness of the results. Additionally, the assessment of sugar intake was based on overall consumption without distinguishing between sources, such as naturally occurring sugars in foods versus added sugars in beverages, limiting our ability to draw specific conclusions about their differential impacts. Finally, the relatively small sample size may restrict the generalizability of the findings, suggesting that further research with larger cohorts is necessary to validate these results and explore the nuances of these relationships more comprehensively.

## 5. Conclusions

This study highlights the significant relationships between sugar consumption, QoL, and depression in breast cancer patients undergoing treatment. High sugar intake may negatively impact QoL and contribute to depressive symptoms. Mindfulness and savoring appear to buffer these effects, suggesting their potential as psychological interventions to enhance emotional resilience. Future studies might explore the long-term impacts of sugar consumption on depression, and integrating nutrition and mindfulness-based support into a depression program for women receiving active breast cancer treatment.

## Figures and Tables

**Table 1:** Demographic and clinical characteristics (n = 78).

Variables	Mean (SD)/[min, max]	n (%)
Age (year)	49.95 (9.80)/[32, 78]	
Education level		
High school and below		12 (15.38)
College and above		66 (84.62)
Working status		
Unemployed		23 (29.49)
Employed		55 (70.51)
BMI (kg/m^2^)	22.70 (3.24)/[18.13, 35.34]	
Cancer stage		
0		4 (5.13)
I		20 (25.64)
II		40 (51.28)
III		14 (17.95)
Mastectomy		45 (57.69)
Chemotherapy		62 (79.49)
Radiotherapy		10 (12.82)
Comorbidity of other physical disease		28 (35.90)

Note. BMI, Body Mass Index.

**Table 2:** Outcomes and macronutrients (n = 78).

Variables	Mean (SD)/[min, max]	n (%)
Depression	6.45 (4.83)/[0.00, 24.00]	
Below clinical depression cutoff		64 (82.05)
Meets clinical depression cutoff		14 (17.95)
Global health status	59.29 (20.67)/[0.00, 100.00]	
QLQ-C30 functions	75.52 (14.26)/[38.67, 100.00]	
QLQ-C30 symptoms	25.16 (14.39)/[0.00, 64.81]	
QLQ-BR45 functions	29.39 (14.49)/[0.00, 60.00]	
QLQ-BR45 symptoms	26.85 (14.20)/[2.46, 67.53]	
Mindfulness—Total score	54.08 (7.02)/[40.00, 73.00]	
Observing	12.12 (2.12)/[6.00, 15.00]	
Describing	11.31 (2.08)/[6.00, 15.00]	
Acting with awareness	11.28 (2.13)/[7.00, 15.00]	
Non-judging	8.56 (2.63)/[3.00, 15.00]	
Non-reactivity	10.81 (2.47)/[3.00, 15.00]	
Savoring: During events	7.17 (1.26)/[4.00, 10.00]	
Savoring: After events	8.36 (1.24)/[5.00, 10.00]	
Physical activity (MET-h/week)	22.90 (30.21)/[0.00, 170.00]	
Sedentary (h/day)	7.76 (3.33)/[0.00, 16.00]	
Macronutrients		
Energy (kcal)	1649.01 (359.54)/[838.98, 2547.32]	
Protein (% Energy)	25.62 (7.65)/[7.89, 48.47]	
Total Fat (% Energy)	36.28 (10.68)/[16.13, 72.74]	
Carbohydrate (% Energy)	44.73 (12.86)/[23.91, 87.98]	
Sugar (% Energy)	14.61 (5.28)/[3.70, 27.05]	
Sugars intake ≤ 10% Energy		13 (16.67)
Sugars intake < 50 g		30 (38.46)

Note. QLQ-C30, Quality of Life Questionnaire for Cancer; QLQ-BR45, Quality of Life Questionnaire for Breast Cancer; MET, Metabolic Equivalent of Task.

**Table 3:** Multivariable linear regression models with depression measured by PHQ-9 (n = 78).

	Univariable		Model 1		Sugar (g)		Sugar (% Energy)	
	*B* (95% CI)	*p*-Value	*B* (95% CI)	*p*-Value	*B* (95% CI)	*p*-Value	*B* (95% CI)	*p*-Value
Sugar (g)	0.12 (0.07 to 0.18)	<0.0001			0.04 (−0.00 to 0.08)	0.18		
Sugar (% Energy)	0.35 (0.16 to 0.54)	<0.001					0.09 (−0.04 to 0.22)	0.08
Stage	1.44 (0.07 to 2.81)	0.04	0.24 (−0.63 to 1.10)	0.59	0.19 (−0.67 to 1.04)	0.58	0.24 (−0.63 to 1.10)	0.66
QLQ-C30 functions	−0.25 (−0.30 to −0.20)	<0.0001	−0.12 (−0.21 to −0.04)	<0.01	−0.11 (−0.20 to −0.03)	0.01	−0.11 (−0.20 to −0.03)	0.01
QLQ-C30 symptoms	0.25 (0.20 to 0.30)	<0.0001	0.05 (−0.05 to 0.14)	0.31	0.04 (−0.05 to 0.13)	0.40	0.04 (−0.05 to 0.13)	0.40
QLQ-BR45 functions	−0.20 (−0.26 to −0.13)	<0.0001	−0.07 (−0.13 to −0.02)	<0.01	−0.08 (−0.13 to −0.02)	0.01	−0.07 (−0.13 to −0.02)	0.01
QLQ-BR45 symptoms	0.24 (0.18 to 0.29)	<0.0001	0.05 (−0.03 to 0.12)	0.21	0.04 (−0.03 to 0.12)	0.17	0.05 (−0.02 to 0.13)	0.23
Mindfulness total score	−0.22 (−0.27 to −0.19)	<0.0001	−0.01 (−0.13 to 0.10)	0.84	−0.01 (−0.13 to 0.10)	0.71	−0.02 (−0.14 to 0.09)	0.81
Savoring: During events	−0.46 (−1.33 to 0.04)	0.29						
Savoring: After events	−1.61 (−2.42 to −0.79)	<0.001	−0.71 (−1.31 to −0.11)	0.02	−0.72 (−1.31 to −0.13)	0.02	−0.70 (−1.29 to −0.10)	0.02
(Constant)			21.65 (11.07 to 32.23)	<0.001	19.35 (8.62 to 30.08)	<0.001	19.90 (9.07 to 30.74)	<0.001
*F*-test (*p*-value)			23.00 (<0.0001)		21.16 (<0.0001)		20.59 (<0.0001)	
R^2^ (adjusted R^2^)			0.70 (0.67)		0.71 (0.68)		0.70 (0.67)	

Note. QLQ-C30, Quality of Life Questionnaire for Cancer; QLQ-BR45, Quality of Life Questionnaire for Breast Cancer.

## Data Availability

The data supporting the findings of this study are available from the corresponding author upon reasonable request.

## Supplementary Materials

The following supporting information can be downloaded at https://www.mdpi.com/article/10.3390/bs15070940/s1: Table S1: Pearson’s correlation matrix between the study variables (N = 78); Table S2: Simple mediation model analysis (N = 78).
