# Supplementary material for: Factors Influencing the Relationship Between Sugar Consumption and Depression Among Women Under Breast Cancer Treatment
Source: Behav Sci (Basel). 2025 Jul 11;15(7):940. doi: 10.3390/bs15070940 (PMC12292852; doi:10.3390/bs15070940)
Supplement: Supplementary file 1 [file behavsci-15-00940-s001.zip › behavsci-3556998-supplementary.pdf]

Supplementary Files

TABLE S1 Pearson's correlation matrix between the study variables (n=78)

	Mean ± SD	1	2	3	4	5	6	7	8	9	10	11	12	13	14
1. Depression	6.45 ± 4.83	−													
2. Global health status	59.29 ± 20.67	−0.58***	−												
3. QLQ−C30 FUN	75.52 ± 14.26	−0.74***	0.73***	−											
4. QLQ−C30 SYM	25.16 ± 14.39	0.75***	−0.78***	−0.83***	−										
5. QLQ−BR45 FUN	29.39 ± 14.49	−0.59***	0.50***	0.44***	−0.56***	−									
6. QLQ−BR45 SYM	26.79 ± 14.27	0.70***	−0.60***	−0.71***	0.77***	−0.55***	−								
7. FFMQ−TS	54.08 ± 7.02	−0.48***	0.44***	0.44***	−0.47***	0.35**	−0.40***	−							
8. FFMQ−OBS	12.12 ± 2.12	−0.10	0.32**	0.23*	−0.34**	0.10	−0.19	0.66***	−						
9. FFMQ−DES	11.31 ± 2.08	−0.41***	0.41***	0.38***	−0.38***	0.24*	−0.33**	0.71***	0.47***	−					
10. FFMQ−AA	11.28 ± 2.13	−0.43***	0.32**	0.44***	−0.33***	0.23*	−0.31**	0.70***	0.30**	0.45***	−				
11. FFMQ−NJ	8.56 ± 2.63	−0.25*	0.41***	0.06	−0.08	0.25*	−0.17	0.35**	−0.14	−0.03	0.15	−			
12. FFMQ−NR	10.81 ± 2.47	−0.29**	0.07	0.30**	−0.36**	0.25*	−0.26*	0.71***	0.52***	0.41***	0.35**	−0.07	−		
13. Savoring: During events	7.17 ± 1.26	−0.12	0.25*	0.08	0.01	0.12	0.01	0.20	0.10	0.10	0.33**	−0.11	0.24*	−	
14. Savoring: After events	8.36 ± 1.24	−0.41***	0.27*	0.27*	−0.32**	0.18	−0.26*	0.51***	0.45***	0.36**	0.27*	0.04	0.50***	0.23*	−
**Macronutrients**															
Sugar (g)	57.40 ± 17.59	0.45***	−0.46***	−0.44***	0.43***	−0.21	0.38***	−0.19	0.02	−0.27*	−0.20	−0.10	−0.06	0.11	−0.11
Sugar (% Energy)	14.61 ± 5.28	0.38***	−0.48***	−0.42***	0.38***	−0.17	0.26*	−0.09	0.04	−0.20	−0.03	−0.06	−0.02	0.08	−0.10

Note. *, $p < .05$; **, $p < .01$; ***, $p < .001$; QLQ−C30, Quality of Life Questionnaire for cancer; FUN, functions; SYM, symptoms; QLQ−BR45, Quality of Life Questionnaire for Breast Cancer, FFMQ, Five Facet Mindfulness Questionnaire; TS, total score; OBS, Observing; AA, Acting with Awareness; NJ, Nonjudging; NR, Nonreactivity.

Table S2 Simple mediation model analysis (n=78)

	Mediator: QLQ-C30 functions		Mediator: QLQ-C30 symptoms		Mediator: QLQ-BR45 symptoms	
Variables	*B* (95% CI)	*p*-value	*B* (95% CI)	*p*-value	*B* (95% CI)	*p*-value
Direct effects						
Sugar (%Energy)-mediator	−1.13 (−1.69, −0.57)	<0.001	1.05 (0.47, 1.62)	<0.001	0.71 (0.12, 1.30)	0.02
Mediator-Depression	−0.24 (−0.30. −0.18)	<0.0001	0.24 (0.18, 0.29)	<0.0001	0.22 (0.16, 0.27)	<0.0001
Sugar (%Energy)-Depression	−0.08 (−0.08, 0.23)	0.31	0.10 (−0.05, 0.25)	0.18	0.19 (0.04, 0.34)	0.01
Total effect	0.35 (0.16, 0.54)	<0.001	0.35 (0.16, 0.54)	<0.001	0.35 (0.16, 0.54)	<0.001
Indirect effect: Sugar (%Energy)-mediator-Depression	0.27 (0.13, 0.44)	<0.001	0.25 (0.11, 0.41)	<0.001	0.15 (0.03, 0.30)	0.02
